# Personalized Nursing: How Life Satisfaction and Occupational Burnout Influence New Competences of Polish Nurses

**DOI:** 10.3390/jpm10020048

**Published:** 2020-06-08

**Authors:** Anna Bartosiewicz, Edyta Łuszczki, Katarzyna Dereń

**Affiliations:** 1 Institute of Health Sciences, Medical College of Rzeszow University, 35-959 Rzeszow, Poland; eluszczki@ur.edu.pl (E.Ł.); kderen@ur.edu.pl (K.D.); 2 Centre for Innovative Research in Medical and Natural Sciences, University of Rzeszow, 35-310 Rzeszow, Poland

**Keywords:** nurse, occupational burnout, prescribing, satisfaction with life

## Abstract

Nursing around the world is developing very dynamically and nurses are undertaking increasingly complex tasks. The extension of entitlements for nurses in Poland in the area of writing prescriptions and referrals for diagnostic tests seems to be a response to the development and changes occurring in this profession. This will improve the standards of patient care, increase access to medical services and improve the professional status of this group. The aim of this study was to analyze the opinions of nurses regarding their preparedness for administering prescriptions and referrals for diagnostic tests depending on their sense of life satisfaction and the level of occupational burnout. The study was conducted among primary care nurses using a survey technique, using a standardized scale of life satisfaction and a scale to measure burnout. In addition, this study used a proprietary survey questionnaire containing questions regarding the self-assessment of preparedness for new competences. The results showed that nurses do not feel well prepared for new tasks. The levels of life satisfaction and burnout of the nurses surveyed significantly influenced confidence regarding their preparedness for writing prescriptions and referrals for diagnostic tests. Polish nurses have a very cautious attitude towards new competences. However, this is a breakthrough and the first step towards approving the role of an advanced practice nurse in our country.

## 1. Introduction

The development of nursing and the assumption of new functions by nurses have meant that nursing has become a more complex profession in many countries, requiring experience and the necessary knowledge to execute and manage patient care [1]. The introduction of new entitlements for nurses in Poland in 2016 in the field of writing prescriptions and referrals for diagnostic tests seems to be the answer to the above-cited changes occurring in this profession [2]. According to the expectations of the International Council of Nurses, this will improve the standards of patient care, and increase access to medical services, while helping to both use the skills of nurses and improve their professional status [3]. The ability of nurses to prescribe prescriptions is a new opportunity in Poland and has raised controversy and concerns. However, according to numerous scientific reports, these powers have been implemented in many countries around the world for a long time and have brought great benefits to their entire healthcare systems [4]. The first country to grant nurses permission to prescribe medicines was the United States in 1960. Other countries, such as Canada, Sweden, England, Australia, New Zealand, the Netherlands, Ireland, Spain and Botswana also have implemented similar measures [3,5]. Pursuant to Polish legislation, a nurse who holds a master’s degree in nursing can only order specific medications, while a nurse with a license can only prescribe medications as a continuation of treatment previously prescribed by a doctor [6]. Nurses who have graduated before attaining these qualifications must have completed a specialist course in this field [7]. The extension of nursing rights resulted in the necessity of introducing changes in nursing teaching standards. Nursing students who started their studies after the introduction of the right to prescribe prescriptions do not have to attend this course, because their study program is significantly extended in this respect [8]. The exact list of medicines and diagnostic tests for which nurses in Poland can write prescriptions is regulated by the ordinance of the Minister of Health [9]. In most Western European and Anglo-Saxon countries, nurses are provided with thorough knowledge related to prescribing during undergraduate and postgraduate courses, yet the related issues are mainly included in regular teaching curricula at the level of master’s degree courses [3,10]. In some countries, such as Ireland and the United Kingdom, nurses acquire the relevant qualifications during independent courses, at bachelor’s level. For instance, in the United Kingdom, the range of medicines which can legally be prescribed by nurses depends on the entitlement category [3,11].

New qualifications for nurses are undoubtedly a great honor and prestige for the profession, but they also are a huge responsibility. They require not only knowledge and substantive skills from the nurses, but also good mental condition [5,12,13]. Work is an important element in everyone’s life and affects their functioning in everyday life. It can be a source of many joys and successes, as well as a reason for breakdowns and depression [14]. The work of a nurse has a special dimension. It is like a mission that on the one hand brings a lot of joy and satisfaction, but can also be a reason for experiencing many difficulties and dilemmas [15]. As numerous studies show, burnout is common among nurses [16]. Polish legislation granting new competences to nurses does not oblige them to accept them, but it gives such an opportunity [2]. The choice of whether to accept them will largely depend on individual willingness to supplement knowledge in the required scope, as well as on individual condition and mental resilience. The right working environment and appropriate support will help overcome all uncertainties and facilitate decisions related to undertaking new tasks [17]. The main reason for the undertaking of this research was the introduction of new entitlements for nurses by Polish legislation. This is a new topic in our country that raises a lot of controversy and criticism. Addressing this topic in scientific research can help to determine whether the addition of nursing competences is the right action.

The main purpose of this study was to examine how nurses assess their preparedness for writing prescriptions and referrals for diagnostic tests, depending on life satisfaction and burnout level.

## 2. Results

### 2.1. Characteristics of the Studied Group

The average age of nurses participating was 47.76 ± 9.65 years and ranged from 22 to 69 years. Over half of the respondents (411; 54.4%) had worked in the nursing profession for over 20 years. More than one-fifth of the nurses (159; 21.0%) had been employed from 16 to 20 years. Fewer nurses had worked in the profession for 1–5 years (47; 6.2%), from 6–10 years (68; 9.0%) or from 11–15 years (71; 9.4%). Almost half of the respondents (343; 45.4%) were nurses with secondary nursing education. Nurses with a professional bachelor’s degree in nursing were the second most numerous, amounting to 157 (20.8%) respondents. The third group was that of the 104 (13.8%) nurses with a master’s degree. The majority of nurses participating in the study (N = 661 i.e., 87.4%) had additional qualifications, i.e., specializations and various further training courses. The nurses declared that they had an employment contract at a public healthcare facility which was their primary and only workplace.

The ages of the respondents differentiated the readiness of the respondents to take on new competences. It was shown that older nurses were less ready to prescribe strong drugs (rho = −0.158; *p* < 0.0001) than younger nurses, who in turn indicated a greater preparedness for prescribing narcotic drugs (rho = −0.100), psychotropic drugs (rho = −0.118; *p* = 0.0011) and antibiotics (rho = −0.130; *p* = 0.0003) than older nurses. The age of the respondents did not significantly affect the level of preparedness for prescribing other drugs. Nurses’ seniority significantly influenced readiness to prescribe selected drugs or agents. It was found that the willingness to prescribe strong drugs decreased along with the increase of the seniority of nurses. It was also found that nurses with a seniority of 1–5 years had greater preparedness for prescribing narcotic drugs (1.83 ± 1.11; *p* = 0.0304), psychotropic drugs (1.98 ± 1.19; *p* = 0.0169) and antibiotics (2.15 ± 1.18; *p* = 0.0003) than their colleagues with more work experience. 

The option to write a referral to all laboratory tests at the nurse’s discretion was more often indicated by older nurses (48.96 years) than younger ones (47.43); the differences in age were not statistically significant, as was the case for seniority. Nurses with master’s degrees declared greater preparedness for writing referrals for ECG tests and wound swabs in comparison to nurses with a lower level of education. Similarly, in the case of having specialization in the field of nursing, nurses declared that they were prepared to write referrals to patients for all laboratory tests at the nurse’s discretion (28.4%) or tests for swabbing from wounds (51.1%).

The level of life satisfaction slightly decreased with the age of nurses (rho = −0.087; *p* = 0.0172), as was the case with seniority. 

On the other hand, nurses with higher education and additional qualifications also had a higher level of life satisfaction (22.41 ± 4.66).

Younger nurses had a higher level of occupational burnout and were characterized by higher scores on the subscales of psychophysical exhaustion (rho = −0.113; *p* = 0.0019) and lack of involvement in relationships (rho = −0.170; *p* < 0.0001). Higher levels of occupational burnout were indicated for nurses who had been working for 1–5 years (14.23 ± 6.51; *p* = 0.0003) and nurses who had been working for 16–20 years (17.29 ± 7.37; *p* = 0.0003). Nurses with a master’s degree and additional qualifications had a higher level of burnout, and this was related to the subscale associated with a lack of commitment to relationships (13.49 ± 4.20; *p* = 0.0091).

### 2.2. Self-Assessment of the Preparedness of Nurses for Writing Prescriptions 

The results obtained show that nurses are not well prepared to take on new competences. The lowest levels of preparedness were declared in the scope of prescribing narcotic drugs, psychotropic drugs and antibiotics, while the greatest ones were indicated for the prescription of drugs previously ordered by a doctor and medical devices (Table 1). 

The use of a 5-point Likert scale (where 1 indicates “definitely not ready” and 5 “definitely ready”) allowed the assessment of the average readiness to prescribe selected drugs in the range of 1–5 points, where higher results corresponded to higher levels of preparedness for prescribing these drugs. 

The analysis of this research showed that nurses had the highest level of preparedness for prescribing medicines in relation to medicines previously ordered by the doctor (3.35 ± 1.47). In second place was the option of prescriptions for medical devices (3.34 ± 1.43), followed by food for special nutritional uses (2.63 ± 1.35) in third place. The subjects showed lesser levels of preparedness for the administration of potent drugs (2.00 ± 1.15) and antibiotics or chemotherapeutics (1.74 ± 1.04). They showed the least preparation for the prescription of psychotropic drugs (1.67 ± 0.99) and narcotic drugs (1.51 ± 0.82). 

A group of 76.9% of respondents (N = 581) decided that a nurse should be able to refer for basic tests (morphology, glucose, urine, ESR). In second place was the possibility of issuing a referral for a blood glucose test (N = 496; 65.6%), and in third place was a referral for a urine test (N = 431; 57.0%). Half of the respondents (N = 379; 50.1%) said that the nurse should have the option of issuing ECG referrals to the patient, while 38.4% (N = 290) indicated the possibility to write a referral for a wound swab. A group of 21.6% of the nurses (N = 163) stated that they should be able to issue referrals for all examinations at their discretion. Few respondents concluded that a nurse should not issue any referrals to the patient (N = 32; 4.2%). Also, a small group of respondents were of the opinion that the nurse should be able to issue a referral for all laboratory tests at the patient’s request (N = 19; 2.5%). Another small group (N = 16; 2.1%) indicated that the nurse should be able to issue a referral for other tests (most often RTG, USG, TSH). The results do not add up to 100% because the respondents were able to indicate more than one answer. 

### 2.3. The Satisfaction with Life (SWLS)

The average result for the life satisfaction scale in the examined group was 19.71 ± 5.41 points, where the results could be in the range of 5–35 points. Half of the respondents obtained no more than 20 points, and 75% of respondents achieved at most 24 points. Among the respondents were nurses who received the lowest possible life satisfaction score and people who received the maximum points (Table 2).

In addition, the results of the life satisfaction scale were divided according to accepted standards, which allowed the conclusion that 33.7% of nurses had a low level of life satisfaction (N = 255). An average level of life satisfaction was indicated by 40.6% of respondents (N = 307), and 25.7% of nurses (N = 194) presented a high level of life satisfaction.

### 2.4. Self-Assessment of Preparedness for Prescribing Medicines and Satisfaction with the Subjects’ Lives

Self-assessment of preparedness for prescribing medicines was significantly associated with life satisfaction. Nurses who had a higher level of life satisfaction rated their preparedness for prescribing foods for particular nutritional uses (rho = 0.095; *p* = 0.0092), medical devices (rho = 0.117; *p* = 0.0012), potent drugs (rho = 0.138; *p* = 0.0001), narcotic drugs (rho = 0.078; *p* = 0.0311) and psychotropic drugs (rho = 0.085; *p* = 0.0196) as better (Table 3).

The level of satisfaction with the life of the surveyed nurses did not significantly affect their self-assessment of preparedness for issuing referrals (Table 4).

### 2.5. The Level of Occupational Burnout—LBQ

The level of occupational burnout included four subscales, out of which the results of each scale could be in the range of 6–36 points. Higher levels of burnout corresponded to higher scores. The highest level of occupational burnout was indicated for psychophysical exhaustion (16.00 ± 6.21). The results of this scale were in the range of 6–36 points, and half of the respondents obtained no more than 15 points. Another category related to occupational burnout was disappointment (15.43 ± 6.68). In the least degree, occupational burnout in nurses was manifested by a lack of commitment to relationships (14.98 ± 5.30) and a sense of lack of professional effectiveness (14.97 ± 3.57) (Table 5).

### 2.6. Self-Assessment of Preparedness for Drug Prescription and the Level of Occupational Burnout

The level of occupational burnout in nurses partly influenced willingness to prescribe medication. It reduced the willingness to the greatest extent in the area of prescription medications previously prescribed by a doctor, because higher confidence in preparedness for taking up new competencies corresponded to higher results in all areas of burnout (rho < 0). It was also shown that occupational burnout significantly reduced nurses’ preparedness for prescription of potent drugs. The area of occupational burnout related to the “disappointment” subscale (rho = −0.131; 0.0003), which negatively affected the preparedness for prescribing medical devices (rho = −0.155; 0.0015), was of the greatest importance here. Correlations between the other criteria of occupational burnout and preparedness for prescribing selected drugs were of little or no significance (Table 6).

It was found that the level of occupational burnout mainly reduced willingness for writing referrals for wound swabs. Nurses with a lower level of lack of commitment to relationships or lower horizontal disappointment were more prepared to write referrals for a wound swab compared to nurses with a higher level of occupational burnout in these dimensions (Table 7).

The analysis showed a correlation between the level of life satisfaction and the level of burnout of the surveyed nurses. Higher levels of life satisfaction were correlated with lower the levels of occupational burnout, especially in the psychophysical exhaustion subscale (Table 8).

## 3. Discussion

Since 2016, nurses in Poland have obtained the ability to prescribe prescriptions and write referrals for diagnostic tests [2]. Over the past decades, more and more countries have granted nurses the right to write prescriptions, with the aim of increasing access to healthcare, especially in regions with insufficient physicians [3,4,5]. Of course, the range of medications for which nurses can write prescriptions varies from country to country and depends on population distribution, the functioning of the healthcare system and the professional status of nurses [18]. Our research has shown that the surveyed nurses do not feel well prepared to undertake new tasks, and this applies in particular to prescribing narcotic drugs, psychotropic drugs and antibiotics. Nurses declared the highest level of preparedness for prescribing medications previously ordered by a doctor, medical devices and foodstuffs for particular nutritional uses, as well as issuing referrals for basic tests, blood glucose tests and urine tests. This may be due to the fact that, for many years, the above-mentioned competences were exclusively the domain of doctors. As recommended by medical circles, emphasis should be placed on proper training of nurses in this field, because the right amount of knowledge will be the basis for the proper fulfilment of new tasks and will also increase the confidence of nurses [7]. As shown by examples from other countries such as Sweden, New Zealand, England, Ireland, the United States and South Africa, including nurses in an interdisciplinary team is beneficial to the entire healthcare system [3,5]. It influences the use of the potential of the whole group of educated nurses and raises the prestige of the profession [18]. In Poland, the lawmakers initially defined some groups of medicines (33 active substances) which can be prescribed by nurses. This is not many, for instance, in comparison to the Swedish list of 230 medicines which can be prescribed to patients by qualified community nurses for 60 medical conditions [9,18]. Nurse prescribers in the United States have full autonomy in prescribing, and this includes intoxicating and psychotropic medication [18,19]. It should be expected that the list of medicines in Poland will also be gradually expanded, but this requires time and, above all, good substantive preparation of nurses.

The analysis of the research showed that life satisfaction among the surveyed nurses was at medium and low levels for the majority, i.e., 74.1%, of the respondents. Similar results were obtained by other researchers examining the level of life satisfaction among nurses. [20,21]. The obtained results confirm the research carried out by the Public Opinion Research Center, which shows that only half of Polish nurses (50%) feel satisfied with life and work; for comparison, it is about 60% in the United States, 62% in Scotland, 64% in England, 67% in Canada and as high as 83% in Germany [21,22,23].

Considering the sense of overall satisfaction with life among nurses, it is necessary to point out the satisfaction associated with the work performed, the sense of professional autonomy and the attitude of the individual towards life and values that make them happy [24]. In Polish and foreign literature, it can be seen that researchers are highly interested in the problem of life satisfaction in relation to job satisfaction among nurses. [24,25]. Our research showed that nurses who had a higher level of life satisfaction rated their preparedness for new competences as better. According to another researcher, satisfaction with life and work is related to readiness to learn and take on new challenges. The author states in his analysis that higher satisfaction with life and work means greater readiness to learn and develop. As other researchers say, the sense of satisfaction among nurses is a strong motivating factor and contributes to greater involvement in the work performed [26,27]. This confirms the work of Laguna, which states that satisfaction can become one of the factors that motivate employees to undertake various types of development activities. Her research shows that high job satisfaction is related to undertaking development activities and expanding professional competences [28]. This is associated with a sense of greater confidence and a greater willingness to take on new challenges, as opposed to people who have lost their joy of life and feel that each new task is beyond their means [28,29].

The problem of occupational burnout among nurses is often taken up by researchers due to the specificity of work in this profession [30]. Intensive and constant involvement in working with people and accompanying people with a disease provide greater exposure to occupational burnout among this professional group [31,32]. Our research has shown that the highest level of occupational burnout was related to psychophysical exhaustion and disappointment; occupational burnout in nurses was manifested to the least extent by a lack of involvement in relationships and a sense of lack of professional effectiveness. A similar level of occupational burnout among nurses is presented in the results of literature analysis in this area over recent years [30,32]. The level of occupational burnout of nurses partly influenced willingness to prescribe drugs, especially in the areas of prescribing medications previously prescribed by a doctor, medical devices and powerful drugs. This was most evident in the disappointment subscale. However, in the case of issuing referrals for diagnostic tests, the level of occupational burnout mainly reduced the confidence in issuing referrals for a wound swab. Occupational burnout can cause reluctance to take on new challenges, such as in the case of writing of prescriptions by nurses. According to Jaworowska, a discouraged person loses his passion and enthusiasm for doing his job and for undertaking new tasks [33]. As Maslach and Sęk say, people with occupational burnout can be observed engaging in different ways of withdrawing from work activity or performing unnecessary activities without the chance to take on new challenges [34,35,36]. People with burnout syndrome are characterized by a minimalist attitude, consisting in performing only the necessary activities, that adversely affects professional development, work efficiency and the quality of services provided [28,37]. 

Our study has some limitations. They mainly result from the fact that it was conducted shortly after the introduction of new competences for nurses. Thus, not all of them had managed to participate in courses and training sessions preparing them for writing prescriptions and referrals for diagnostic tests for patients, which in turn may have resulted in affecting their attitude and lowering their self-esteem in the presented results. In addition, only women were included in the study group. The nursing profession in Poland is very feminized, and less than 2% in this profession are male. They usually work in intensive care units, operating theaters and treatment departments, meaning that access to them is significantly limited. We plan to repeat this research on a large group of nurses (including men) using standardized questionnaires, which will additionally check the quality of these tasks (writing prescriptions and referrals for diagnostic tests for patients) and indicate areas that should be improved.

## 4. Materials and Methods

The research was conducted in 2017, considering primary healthcare nurses in Southeastern Poland, namely the Podkarpackie region. We sent invitations to participate in the study to 37 randomly selected primary healthcare centers in Southeastern Poland, containing a letter detailing the purpose of the study, a consent form and a questionnaire. Thirteen centers responded and agreed to participate in the study. 

Nurses who were working in primary healthcare in the Subcarpathian Province (Southeastern Poland), who were registered with the District Council of Nurses, who had the right to practice, who had at least 2 years of professional experience, who were present during data collection, who were willing to participate in the study and who agreed to participate in the study were considered as eligible for inclusion.

Nurses who were not working with primary healthcare medical entities, nurses who were not registered with the District Council of Nurses, nurses who did not have the right to practice, nurses who had less than 2 years of work experience, nurses who did not agree to participate in the study, nurses who resigned from participating in the study at any stage and nurses who incorrectly completed their questionnaires were excluded from the study.

The survey questionnaires with the consent forms were delivered to the primary healthcare units.

Participation in the study was voluntary and anonymous. The subjects first received a verbal description of the purpose and its voluntary nature. The subjects were given assurance that their agreement or refusal to participate would not impact their further employment at the relevant healthcare entity. To ensure confidentiality of the data, the questionnaires were labeled with numbers. The completed questionnaires were received by the study organizers in sealed envelopes attached to the questionnaire. Correctly completed questionnaires were equivalent to the subjects’ consent to participate in the study. A total of 1320 surveys were distributed, of which 800 (60%) were collected back. After checking, 44 questionnaires were rejected due to the incompleteness of the answers given. Data from the remaining 756 surveys were analyzed using statistical methods. The examined nurses (756) were a representative sample (25%) of all nurses (N = 3025) employed in primary healthcare sector of this region of Poland. The study group consisted of 756 nurses from primary healthcare who were professionally active and had at least 2 years of professional experience. In addition, 146 (i.e., 19.3%) of the nurses surveyed additionally held the position of health visitor, 76 (i.e., 10%) held the position of coordinating nurse, 28 (i.e., 3.7%) worked in the teaching and upbringing (school) environment and 515 (i.e., 68%) worked in primary healthcare entities in the treatment room and vaccination point (as so-called cooperating nurses). The research method used in this work was a diagnostic survey conducted using a survey technique.

The participants completed the questionnaire containing seven questions concerning sociodemographic data, i.e., age, seniority, education (secondary, bachelor’s, master’s); additional qualifications (specialization); and form of employment (temporary or permanent contract). Further questions were more extensive and were related to the assessment of self-preparedness for writing prescriptions and referrals for diagnostic tests. The respondents could select answers from the following choices: strongly disagree, disagree, neither agree nor disagree, agree, strongly agree. Two components have been distinguished, which together account for 69.2% of the variance in total (the first explains 48.7% of the variance and the other explains 20.5% of the variance, together giving a satisfactory result of almost 70% of the explained variance).

The pilot study was conducted using the survey on new competences and Satisfaction With Life Scale (SWLS) on a group of 34 primary healthcare nurses. The aim of the study was to check the correctness of completed questionnaires in terms of understanding the questionnaire. The average age of the respondents was 38.32 ± 9.81 years (23–55 years); more than half of them were married (58.8%), people with at most first degree studies and specialization (64.7%) and urban residents (58.8%). The reliability of the SWLS was high (Cronbach’s alpha of 0.891), and all scale questions correlated positively with each other (Pearson ratio 0.529–0.797) as well as with the overall scale score (Pearson ratio 0.636–0.808). The obtained results indicate a good understanding of the tool.

In the main research, we used two standardized questionnaires, namely the Satisfaction With Life Scale (SWLS) and Occupational Burnout Questionnaire (LBQ).

The Satisfaction With Life Scale (SWLS), developed by E. Diener, R. A. Emmons, R. J. Larson and S. Griffin), contains five statements evaluated on a 7-point scale. The respondent assesses to what extent each statement relates to his or her current life. The subject of the measurement is the assessment of life satisfaction, which is the result of comparing one’s own satisfaction with the standards they have set. The points are added together. The obtained results, ranging from 5 to 35, determine the degree of satisfaction with life: scores of 5–16 points indicate low results, scores of 17–23 points indicate average results and scores of 24–35 points indicate high results. The Cronbach’s alpha reliability index was satisfactory and amounted to 0.81. The scale is an adaptation of the Polish version [38]. 

The Occupational Burnout Questionnaire (LBQ) is a Polish adaptation of the Italian Link Burnout Questionnaire developed by Massimo Santinello. The publisher is the Psychological Test Laboratory of the Polish Psychological Association in Warsaw, and it was published in 2014. The LBQ is designed to measure occupational burnout in people whose work is related to helping other people and teaching. This questionnaire contains 24 statements divided into four subscales, allowing for the assessment of occupational burnout in four aspects:

- Psychophysical exhaustion—the assessment of one’s psychophysical resources;
- Lack of commitment to customer relationships—this subscale describes the quality of customer/patient relationships;
- Feelings of lack of professional effectiveness—the assessment of one’s own professional competences;
- Disappointment—related to one’s specific motivation to choose a profession related to helping others.

Answers are given on a 6-point adjective scale, whose subsequent points refer to the frequency with which these feelings occur: never, rarely, once or more a month, about every week, several times a week, every day. The obtained results may range from 6–36 points: scores of 6–10 points indicate low results, showing no symptoms of burnout in the subjects; scores of 11–25 points indicate average results, showing the possibility of occurrence of certain problems related to burnout; scores of 26–36 points indicate high results, showing a high level of occupational burnout. The Cronbach’s alpha coefficients of internal compliance for LBQ scale were acceptable and are as follows: psychophysical exhaustion, 0.77; lack of commitment to customer relations, 0.70; sense of ineffectiveness, 0.63; disappointment, 0.84. The coefficient of internal compliance for the LBQ scale is acceptable (varimax rotation) and explained 50.98% of variance, which is a satisfactory result; a comparable result was obtained by the author of the questionnaire. 

LBQ is used in the diagnosis of employee teams, departments and organizations, as well as for preventive purposes to monitor burnout in groups as well as individuals. It is also used as a supplement to tools for assessing professional functioning [33,39].

The estimation method and the following statistical methods were also used:

1. Differences between the variables measured on the qualitative scale were verified using the χ^2^ independence test, taking the correction for Yates continuity and the results of Fisher’s exact test into account in the 2 × 2 tables.
2. Differences between quantitative variables were examined using the Mann–Whitney test and Kruskal–Wallis test and by calculating Spearman’s rho correlation coefficient. This was due to the lack of normality of quantitative variable distributions (verified by the Kolmogorov–Smirnov and Shapiro–Wilk tests) and the lack of equivalence of the compared groups (verified by the χ^2^ compliance test and the binomial test).
3. For the presentation of statistical data, the descriptive statistics method was used, considering the arithmetic mean (M), whose value determines the average level of a given variable, and standard deviation (SD), a statistical measure of the dispersion of results around the expected value.

The level of significance was set to 5%, and calculations were carried out with the program IBM SPSS Statistics 20.

Ethics approval and consent to participate: the research project was carried out in accordance with the Helsinki Declaration. The study was approved by Bioethics Committee at the Rzeszow University (resolution No. 12/12/2015).

## 5. Conclusions

Polish nurses have a very cautious attitude towards new competences. The level of life satisfaction and burnout of the nurses surveyed significantly influenced confidence regarding their preparedness for writing prescriptions and referrals for diagnostic tests. Nurses with higher satisfaction had lower levels of occupational burnout. 

The work environment and education of nurses should be improved and strengthened, as this will translate into their personal development and bring benefits to the entire healthcare system.

## Figures and Tables

**Table 1:** Nurses’ self-assessed preparedness for prescribing medicines.

Kind of Medicine		Strongly Disagree	Disagree	Neither Agree nor Disagree	Agree	Strongly Agree	M	SD
Foodstuffs for particular nutritional uses	**N**	202	197	107	176	74	2.63	1.35
	**%**	26.7%	26.1%	14.2%	23.3%	9.8%		
Medical products	**N**	130	115	57	276	178	3.34	1.43
	**%**	17.2%	15.2%	7.5%	36.5%	23.5%		
Potent drugs	**N**	333	232	78	85	28	2.00	1.15
	**%**	44.0%	30.7%	10.3%	11.2%	3.7%		
Narcotic drugs	**N**	481	199	45	24	7	1.51	0.82
	**%**	63.6%	26.3%	6.0%	3.2%	0.9%		
Psychotropic medicines	**N**	448	183	58	58	9	1.67	0.99
	**%**	59.3%	24.2%	7.7%	7.7%	1.2%		
Antibiotics	**N**	425	194	57	68	12	1.74	1.04
	**%**	56.2%	25.7%	7.5%	9.0%	1.6%		
Drugs previously ordered by a doctor	**N**	138	102	78	230	208	3.35	1.47
	**%**	18.3%	13.5%	10.3%	30.4%	27.5%		

M: arithmetic mean; SD: standard deviation.

**Table 2:** Satisfaction With Life Scale (SWLS) scores of the nurses.

M (SD)	Min–Max	Q1–Q2 (Me)–Q3
19.71 (5.41)	5–35	16–20–24

Q1: quartile I; Q2 (Me): quartile II (median); Q3: quartile III; M: arithmetic mean; SD: standard deviation.

**Table 3:** Nurses’ self-assessed preparedness for prescribing and their satisfaction with life (as assessed by SWLS).

Type of Medicine	*rho	*p*
Foodstuffs for particular nutritional uses	0.095	0.0092
Medical products	0.117	0.0012
Potent drugs	0.138	0.0001
Narcotic drugs	0.078	0.0311
Psychotropic medicines	0.085	0.0196
Antibiotics	0.065	0.0722
Drugs previously ordered by a doctor	0.027	0.4556

rho: Spearman’s correlation; *p*: statistical significance. * Spearman’s rho correlation coefficient.

**Table 4:** Nurses’ self-assessed preparedness for issuing referrals for diagnostic tests and their satisfaction with life (as assessed by SWLS).

Type of Diagnostic Test	No		Yes		*p*
	M	SD	M	SD	
Basic tests (morphology, glucose, urine)	19.98	4.83	19.62	5.57	0.6954
Urine test	19.79	5.35	19.64	5.46	0.8756
Blood glucose level	19.64	5.22	19.74	5.51	0.6373
All laboratory tests on nurse’s request	19.74	5.30	19.58	5.79	0.9290
All laboratory tests on patient’s request	19.67	5.38	20.95	6.41	0.3151
Electrocardiogram	19.95	5.35	19.46	5.46	0.1326
Wound swab	19.59	5.40	19.88	5.43	0.5552
Others	19.72	5.36	19.13	7.68	0.6752
None	19.66	5.45	20.69	4.41	0.4402

M: arithmetic mean; SD: standard deviation; *p*: statistical significance.

**Table 5:** The level of occupational burnout in nurses, as assessed by the Occupational Burnout Questionnaire (LBQ).

LBQ	M (SD)	Min.–Max.	Q1-Q2 (Me)-Q3
Psychophysical exhaustion	16.00 (6.21)	6–36	11-15-20
Lack of commitment to professional relationships	14.98 (5.30)	6–35	11-14-18
The feelings of lack of professional effectiveness	14.97 (3.57)	7–29	13-14-17
Disappointment	15.43 (6.68)	6–34	10-15-20

Q1: quartile I; Q2 (Me): quartile II (median); Q3: quartile III; M: arithmetic mean; SD: standard deviation.

**Table 6:** Nurses’ self-assessed preparedness for prescribing and their level of occupational burnout (as assessed by LBQ).

Type of Medicine	Psychophysical Exhaustion		Lack of Commitment to Professional Relationships		The Feelings of Lack of Professional Effectiveness		Disappointment	
	*rho	*p*	*rho	*p*	*rho	*p*	*rho	*p*
Foodstuffs for particular nutritional uses	−0.025	0.4991	−0.086	0.0182	−0.044	0.2322	−0.066	0.0684
Medical products	−0.085	0.0189	−0.107	0.0031	−0.036	0.3229	−0.115	0.0015
Potent drugs	−0.115	0.0016	−0.077	0.0344	−0.09	0.0137	−0.131	0.0003
Narcotic drugs	−0.026	0.4797	0.001	0.9718	−0.049	0.177	−0.064	0.0781
Psychotropic medicines	−0.046	0.2099	−0.059	0.1054	−0.063	0.083	−0.081	0.0263
Antibiotics	−0.034	0.3538	−0.001	0.984	−0.036	0.3273	−0.067	0.0655
Drugs previously ordered by a doctor	−0.079	0.0295	−0.166	<0.0001	−0.099	0.0064	−0.124	0.0006

rho: Spearman’s correlation; *p*: statistical significance. * Spearman’s rho correlation coefficient.

**Table 7:** Nurses self-assessed preparedness for writing referrals for diagnostic tests and their level of occupational burnout (as assessed by LBQ).

Tests and LBQ	PE				LCPR				FLPE				D			
	No		Yes		No		Yes		No		Yes		No		Yes	
	M	SD	M	SD	M	SD	M	SD	M	SD	M	SD	M	SD	M	SD
**BT**	16.42	6.17	15.87	6.22	15.26	5.57	14.90	5.22	15.35	3.64	14.85	3.54	16.06	6.78	15.24	6.64
*p*	0.2245				0.5081				0.0837				0.1424			
**UT**	16.43	6.23	15.67	6.18	15.44	5.25	14.64	5.32	15.02	3.77	14.92	3.41	16.15	6.98	14.89	6.40
*p*	0.0637				0.0141				0.9373				0.0158			
**BGL**	16.53	6.29	15.71	6.15	15.38	5.26	14.77	5.32	14.83	3.56	15.03	3.57	16.24	6.86	15.00	6.55
*p*	0.0755				0.0610				0.5090				0.0155			
**LTNR**	16.10	6.15	15.63	6.44	15.02	5.20	14.83	5.68	14.95	3.59	15.01	3.50	15.44	6.61	15.40	6.93
*p*	0.2688				0.3844				0.8357				0.7560			
**LTPR**	16.03	6.25	14.74	4.13	15.02	5.34	13.63	3.58	14.95	3.56	15.68	3.70	15.48	6.73	13.32	3.94
*p*	0.7295				0.4292				0.2832				0.2353			
**E**	16.31	6.53	15.68	5.86	15.33	5.44	14.64	5.15	15.01	3.59	14.92	3.54	15.99	6.90	14.87	6.41
*p*	0.3588				0.1023				0.5558				0.0314			
**WS**	16.15	6.18	15.74	6.26	15.48	5.40	14.19	5.04	15.03	3.58	14.86	3.54	16.16	6.69	14.25	6.49
*p*	0.2826				0.0004				0.4457				0.0001			
**O**	16.00	6.21	15.88	6.23	15.01	5.32	13.63	4.30	14.95	3.57	15.81	3.41	15.43	6.65	15.31	8.14
*p*	0.9377				0.3456				0.2399				0.7672			
**N**	15.91	6.13	17.91	7.68	14.98	5.29	14.97	5.69	14.97	3.59	14.97	3.14	15.44	6.66	15.25	7.20
*p*	0.2053				0.9907				0.3874				0.7678			

PE: Psychophysical exhaustion; LCPR: lack of commitment to professional relationships; FLPE: the feelings of lack of professional effectiveness; D: disappointment; BT: basic tests (morphology, glucose, urine); UT: urine test; BGL: blood glucose level; LTNR: all laboratory tests on nurse’s request; LTPR: all laboratory tests on patient’s request; E: electrocardiogram; WS: wound swab; O: others; N: none; M: arithmetic mean; SD: standard deviation; p: statistical significance. Mann–Whitney test was used to calculate the results.

**Table 8:** Satisfaction with life and the level of occupational burnout among nurses (SWLS vs. LBQ).

SWLS and LBQ	Psychophysical Exhaustion	Lack of Commitment to Professional Relationships	The Feelings of Lack of Professional Effectiveness	Disappointment
*rho	−0.224	−0.134	−0.112	−0.221
*p*	0.0000	0.0002	0.0021	0.0000
N	756	756	756	756

* Spearman’s rho correlation coefficient; *p*: statistical significance.

## Data Availability

The data analyzed in this study are available upon request to the corresponding author: abartosiewicz@ur.edu.pl.
